# Synthesis and Ion-Exchange Properties of Graphene Th(IV) Phosphate Composite Cation Exchanger: Its Applications in the Selective Separation of Lead Metal Ions

**DOI:** 10.3390/ijerph14070828

**Published:** 2017-07-24

**Authors:** Tauseef Ahmad Rangreez, Inamuddin, Abdullah M. Asiri, Basma G. Alhogbi, Mu. Naushad

**Affiliations:** 1 Advanced Functional Materials Laboratory, Department of Applied Chemistry, Faculty of Engineering and Technology, Aligarh Muslim University, Aligarh 202002, India; tauseefjh@gmail.com; 2 Chemistry Department, Faculty of Science, King Abdulaziz University, Jeddah 21589, Saudi Arabia; ceamr2@gmail.com (A.M.A.); alhogbib@gmail.com (B.G.A.); 3 Centre of Excellence for Advanced Materials Research, King Abdulaziz University, Jeddah 21589, Saudi Arabia; 4 Department of Chemistry, College of Science, Building 5, King Saud University, Riyadh 11564, Saudi Arabia; shad123@gmail.com

**Keywords:** composite cation-exchanger, graphene Th(IV) phosphate, ion-exchange capacity, nano-composite, selectivity studies

## Abstract

In this study, graphene Th(IV) phosphate was prepared by sol–gel precipitation method. The ion-exchange behavior of this cation-exchanger was studied by investigating properties like ion-exchange capacity for various metal ions, the effect of eluent concentration, elution behavior, and thermal effect on ion-exchange capacity (IEC). Several physicochemical properties as Fourier transform infrared (FTIR) spectroscopy, X-ray diffraction (XRD) study, thermal studies, scanning electron microscopy (SEM) and transmission electron microscopy (TEM) studies were also carried out. The material possessed an IEC of 1.56 meq·dry·g^−1^ of the exchanger and was found to be nano-composite. The selectivity studies showed that the material is selective towards Pb(II) ions. The selectivity of this cation-exchanger was demonstrated in the binary separation of Pb(II) ions from mixture with other metal ions. The recovery was found to be both quantitative and reproducible.

## 1. Introduction

The rapid growth of industry and technology has improved the standard of life, but at the same time it has created a complicated web of environmental pollution [1,2,3]. In order to meet the demands of modern society, natural resources get overburdened, producing harmful and toxic materials that must be disposed of. One of the major threats is that of toxic metals that are released into the environment through anthropogenic activities [4]. Most of the heavy toxic metals from industries, factories, and agricultural use end up in water streams [5]. The presence of these heavy metal ions renders water useless for drinking, owing to their toxic and bio-accumulative nature. Therefore, the detection and removal of these pollutants is of utmost importance in order to get safe potable water.

Lead is considered as one such major toxic metal. It is a potent threat to human life and the ecosystem [6]. The main sources of lead production include the manufacture of lead acid batteries, lead pipes, metal processing plants, printing, mining, and tannery [7,8,9,10,11,12,13]. It gets accumulated in the tissues and causes severe renal, reproductive, and central nervous system failure [11,14,15,16,17]. The Indian standard drinking water specification (Second revision) has set the tolerance limit of lead in drinking water as 0.01 mg/L [18]. As a result of these harmful effects, there is a constant need to monitor, separate, and remove the lead from water sources.

Ion-exchange materials have emerged as a viable option for the separation of metal ions compared to chemical precipitation [19], membrane filtration [20], electrochemical, and thermal treatment in terms of simple procedure and cost effectiveness [21,22]. The incorporation of organic conducting polymer into the matrices of multivalent inorganic metal salts such Th(IV), Sn(IV), Zr(IV), Sb(V), etc. has resulted in the development of hybrid ion-exchangers with superior ion-exchange, granulometric, mechanical properties, and higher selectivity for heavy metals [23,24,25,26,27]. Recently, several studies have been reported for the adsorption of Pb(II) ions on tartaric acid-modified graphene oxide [28], L-glutamic acid-functionalized graphene oxide [29], diiodocarbene-modified graphene [30], dibromocarbene-modified graphene [31], and 3D hierarchical flower-like nickel ferrite/manganese dioxide [32]. In this study, a graphene Th(IV) phosphate composite cation-exchanger was synthesized and was evaluated for its ion-exchange properties. The ion-exchange properties are useful to determine the column efficiency, thermal stability, optimum eluent concentration, as well as separation capability of the ion exchange material. The hybrid cation-exchanger was also used for the separation of Pb(II) ions from a mixture of other heavy metal ions.

## 2. Materials and Methods

### 2.1. Reagents and Instruments

The main reagents used in the synthesis were thorium nitrate (Th(NO_3_)_4_·5H_2_O), orthophosphoric acid (H_3_PO_4_), nitric acid (HNO_3_), and N-cetyl-N,N,N-trimethylammonium bromide (CTAB). Nitrate salts of all metals were used. These reagents and chemicals were purchased from Central Drug House, India. Graphene oxide powder (˂20 µm) with 98% purity was purchased from Sigma Chemicals, India. All reagents and chemicals were of analytical grade. A digital muffle furnace (Macro Scientific Works, MSW-251, Thane, India), digital balance (MAB 220, Rajasthan, Wensar India), and a magnetic stirrer (LMMS-1L4P, LABMAN Scientific Instruments) were used.

### 2.2. Preparation of the Reagent Solutions

Thorium nitrate (0.1 M) was prepared in 1 M HNO_3_, while orthophosphoric acid (2 M) was prepared in demineralized water (DMW). A dispersion of graphene was prepared in CTAB (1 mmol).

### 2.3. Synthesis of Composite Cation-Exchanger

**Scheme 1:**
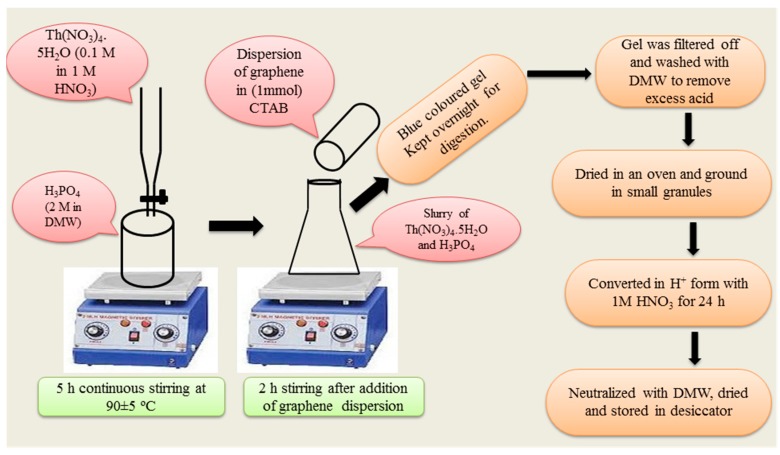
Synthesis of composite cation exchanger. DMW: demineralized water.

The inorganic precipitates of thorium phosphate were obtained by adding a solution of (0.1 M) thorium nitrate prepared in 1 M nitric acid slowly at a flow rate of 0.5 mL/min to a solution of (2 M) H_3_PO_4_ and continuously stirring using a magnetic stirrer at a temperature of 90 ± 5 °C. The resulting white slurry was stirred for 5 h at this temperature. Further, a dispersion of graphene was added to the slurry and stirred for 3 h. The resultant blue-colored gel was kept overnight at room temperature (25 ± 3 °C) for digestion. The supernatant liquid was decanted, and the gel was filtered off. It was repeatedly washed with demineralized water to remove excess acid. The product was dried in an oven maintained at 40 ± 1 °C. The dried product was ground into small granules and converted to H^+^ ion form by treating with 1 M HNO_3_ for 24 h and occasionally replacing the supernatant liquid with fresh acid. The excess acid was removed after several rounds of washing with DMW, dried at 50 ± 1 °C, and sieved to obtain particles of particular size range of ~125 μm and stored in a desiccator. Hence, a number of samples of graphene Th(IV) phosphate were prepared (Table 1). A scheme for the synthesis of composite cation exchanger is shown in Scheme 1.

### 2.4. Physical Characterization

Scanning electron microscopic images of graphene Th(IV) phosphate composite cation-exchanger were taken by scanning electron microscope (JEOL, JSM, 6510-LV, Kyoto, Japan) at an accelerating voltage of 20 kV. TEM studies were carried out to determine the particle size of graphene Th(IV) phosphate composite cation-exchanger using transmission electron microscope (JEM 2100, JEOL, Kyoto, Japan).

### 2.5. Determination of Ion-Exchange Capacity

The standard column process was used to determine the ion-exchange capacity. A glass column having an internal diameter (i.d.) ~1 cm, fitted with glass wool support at the bottom, was filled with one gram (1 g) of the dry cation-exchanger in the H^+^-form. By maintaining a very slow flow rate (~0.5 mL/min), NaNO_3_ (1 M) as eluent was used to elute the H^+^ ions completely from the cation-exchange column. The ion-exchange capacity in meq·dry·g^−1^ of the exchanger was determined by titrating the effluent against a standard 0.1 M NaOH solution using phenolphthalein indicator. On the basis of Na^+^ ion-exchange capacity, sample S-2 was selected for further studies.

#### 2.5.1. Ion-Exchange Capacity for Alkali and Alkaline Earth Metals

One molar (1 M) solutions of alkali and alkaline earth metal nitrates were used as an eluent to elute the H^+^ ions completely from the cation-exchanger column, maintaining a very slow flow rate (~0.5 mL/min). The effluent was titrated as reported [2].

#### 2.5.2. Thermal Effect on Ion-Exchange Capacity

Drying temperature effect on the ion-exchange capacity was studied at various temperatures in a muffle furnace for 1 h. One gram (1 g) of the composite cation-exchangers (S-2) in the H^+^-form were heated, and after cooling them at room temperature, the Na^+^ ion-exchange capacity of each sample was determined by column process [2].

#### 2.5.3. Effect of Eluent Concentration

By passing a fixed volume (250 mL) of a NaNO_3_ solution of different concentrations (0.2 to 1.6 M), the optimal concentration of the eluent for complete elution of H^+^ ions was found through a column containing one gram of the cation-exchange material (S-2) in the H^+^-form. The effluent for the H^+^ ions eluted out was titrated as reported [2].

#### 2.5.4. Elution Behavior

The efficiency of the column containing one gram of the cation-exchanger (S-2) in H^+^-form was determined by eluting the column with a 1 M NaNO_3_ solution in different 10 mL fractions with minimum flow rate as described above. Each fraction of the effluent was for the H^+^ ions eluted out was titrated as reported [2].

#### 2.5.5. Distribution Studies

The batch method was used to determine the distribution coefficients (K_d_-values) of various metal ions on graphene Th(IV) phosphate. Various composite cation-exchanger beads (200 mg) (S-2) in H^+^ ion form were taken in Erlenmeyer flasks with 20 mL of metal nitrate solutions each in the required medium; for example, demineralized water (DMW), 10^−2^ M HClO_4_, 10^−1^ M HClO_4_, 1 M HClO_4_, 10^−2^ M HNO_3_, 10^−1^ M HNO_3_, 1 M HNO_3_, 10^−2^ M HCl, 10^−1^ M HCl, and 1 M HCl and kept for 24 h with continuous shaking for 6 h in a temperature-controlled incubator shaker at 25 ± 2 °C to attain equilibrium. The initial metal ion concentration was adjusted such that it did not exceed 33% of its total ion-exchange capacity. Titration against standard 0.005 M solution of EDTA gave the metal ions in the solution before and after equilibrium [33]. The distribution coefficient (K_d_) values were calculated using the formula given below:

(1) $$K_{d}=\;\frac{I-F}{F}\;\times \;\frac{V}{M}\;\left(\mathrm{mL}\cdot g^{-1}\right)$$

where I is the initial amount of the metal ion in the solution phase, F is the final amount of the metal ion in the solution phase, V is the volume of the solution (mL), and M is the amount of the exchanger (g).

#### 2.5.6. Separation of Metal Ions

Quantitative binary separations of some metal ions of analytical utility were achieved on graphene Th(IV) phosphate column (S-2). One gram of the cation-exchanger in H^+^-form was used for column separations in a glass tube having an internal diameter of ~0.6 cm. The column was washed thoroughly with DMW, and the mixture of two metal ions having initial concentrations of 0.01 M each to be separated was loaded on it and allowed for 1 h to adsorb the metal ions on the exchanger and then recycled through the column three to four times (at a flow rate of 2–3 drops per minute) to ensure complete adsorption of the mixture on column beads; the separation was achieved by passing a suitable solvent at a flow rate of 1 mL/min through the column as eluent. The metal ions in the effluent were determined quantitatively by EDTA titration [33].

#### 2.5.7. Selective Separation of Metal Ions from a Synthetic Mixture

The selective separation of ions from a mixture of Pb^2+^, Ni^2+^, Cd^2+^, and Mg^2+^ in an aqueous solution containing variable amounts of Pb^2+^ ions was carried by pouring the mixture onto the top of the column and allowing it to adsorb the metal ions on the exchanger for 1 h and then recycling through the column three to four times (at a flow rate of 2–3 drops per minute) until the level of sample solution was just above the material surface. The solution was passed through the column a number of times to ensure complete adsorption of the metal ions. The metal ions in the effluent were determined quantitatively by EDTA titration [33].

## 3. Results and Discussion

In this study, graphene Th(IV) phosphate was prepared by sol–gel precipitation method (Table 1). The graphene provides the mechanical stability to the inorganic cation-exchanger and contributes to enhancing the ion-exchange capacity [23]. The inorganic thorium phosphate possessed the ion-exchange capacity of 1.20 meq·dry·g^−1^, which is lower than that of the composite ion-exchange capacity of 1.56 meq·dry·g^−1^. The enhancement in the ion-exchange capacity results from the binding of graphene with inorganic ion-exchanger, and hence leaching of inorganic ion-exchanger was not prominent. A comparison of the ion-exchange capacity of this hybrid cation-exchanger with those of the already-reported lead-selective composite ion-exchangers in the literature showed that the ion-exchange capacity is comparatively better and hence more selective [23,34,35] (Table 2).

**Figure 1:**
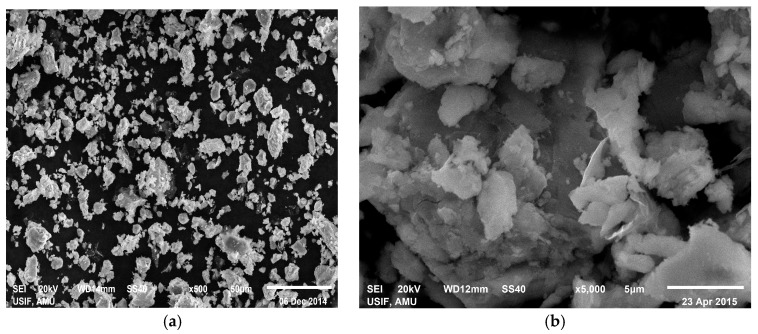
Scanning electron micrograph (SEM) of graphene Th(IV) phosphate cation-exchanger at different magnifications. (**a**) ×500, (**b**) ×5000.

**Figure 2:**
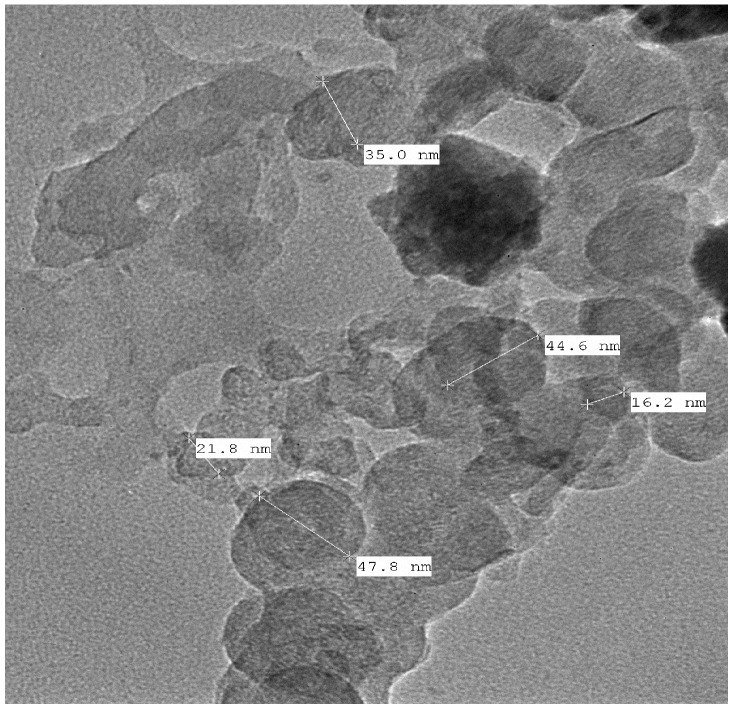
Transmission electron micrograph (TEM) of graphene Th(IV) phosphate cation exchanger.

Fourier transform infra-red (FTIR) spectroscopy, X-ray diffraction (XRD), and simultaneous thermogravimetric analysis (TGA) and differential thermal analysis (DTA) studies of the composite cation exchange material (S-2) have also been carried out [36]. The SEM micrograph of graphene Th(IV) phosphate (Figure 1) shows that the surface of inorganic precipitate of Th(IV) phosphate was uniformly covered with graphene. The organic conducting polymer is tightly binding to the inorganic precipitate, providing mechanical stability and preventing the leaching out of the inorganic precipitate. The TEM studies (Figure 2) reveal that the composite has a varying particle size (i.e., 16.2, 21.8, 35.0, 44.6, and 47.8 nm), and hence the above-prepared material can be considered a nano-composite.

The ion-exchange capacity (IEC) of various exchanging ions on composite cation-exchanger showed that the IEC for alkali and alkaline metal ions increases with the decrease in hydrated ionic radii [37,38] (Table 3). These results are similar with those of Nachod and Wood [39] for the exchange of alkali and alkaline metal ions on carbonaceous zeolites. The effect of temperature on the ion-exchange capacity of graphene Th(IV) phosphate composite cation-exchanger on heating for 1 h showed that the material retained its ion-exchange capacity by heating up to 100 °C. The material retained approximately 64% of its initial ion exchange capacity on heating up to 300 °C. The results indicated that ion-exchange capacity decreases and color appearance of beads changes from light blue to mist gray upon increasing the temperature (Table 4).

**Figure 3:**
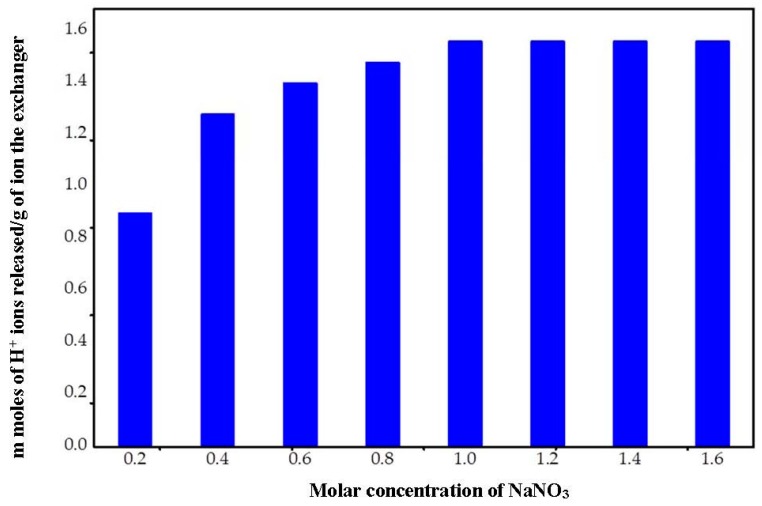
Effect of eluent concentration on the ion-exchange capacity of graphene Th(IV) phosphate cation exchanger.

**Figure 4:**
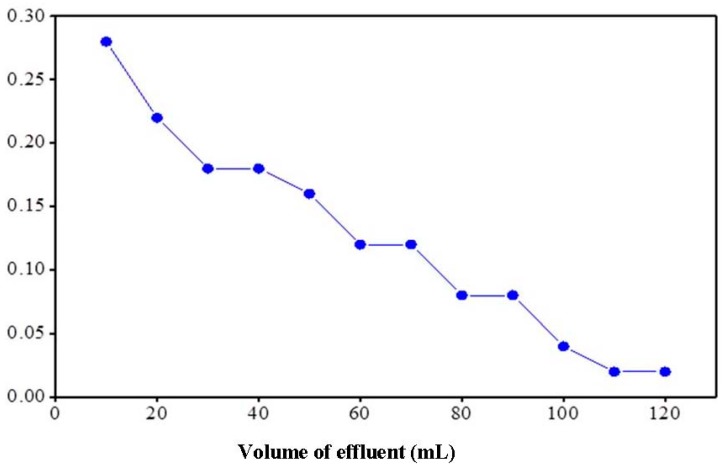
Elution behavior of graphene Th(IV) phosphate cation-exchange material.

The optimum concentration for complete elution of counter/exchangeable ions determined was found to be 1 M metal nitrate solution and indicated that it is sufficient for complete elution of the H^+^ ions (Figure 3). The selected concentration is used to find out the optimum volume of the eluent to elute the H^+^ ions completely as a measure of the column efficiency. The results showed that only 120 mL of the eluent was required to elute the H^+^ ions completely from the cation-exchange column, which indicates good column efficiency (Figure 4).

To demonstrate the analytical utility of this composite cation-exchanger, distribution studies were carried out in various solvent systems of environmental importance (Table 5). It was observed from the higher K_d_-values that the lead is highly adsorbed as compared to other metal ions under study. It was also observed that K_d_-values or selectivity is dependent on the nature and composition of contacting solvents. The separation capability of this cation-exchanger was also demonstrated in the binary separation of lead from a mixture of the lead with other metal ions (Table 6). The weakly adsorbed metal ions elute first from the column, while the strongly held ions were eluted last. It was observed that the recovery of lead was found to be quantitative and reproducible by three replicate measurements. The practical applicability of these separations was demonstrated by employing the composite material in the separation of metal ions from a synthetic mixture of Pb^2+^, Ni^2+^, Cd^2+^, and Mg^2+^ (Table 7).

## 4. Conclusions

In the present study, a Pb^2+^ ion-selective nano-composite ion-exchanger graphene Th(IV) phosphate having an IEC of 1.56 meq·dry·g^−1^ of exchanger was prepared. The TEM analysis revealed that the particle size of the prepared composites was in the range of 16–45 nm. Thus, the cation-exchanger can be considered as a nano-composite material. The composite material was successfully used in the separation of Pb^2+^ ions quantitatively from a synthetic mixture of various metal ions. The environmental monitoring requires quick detection as well as effective separation of pollutants, and hence the selective behavior of this cation-exchanger can be of great significance.

## Figures and Tables

**Table 1:** Conditions of preparation and the ion-exchange capacity of graphene Th(IV) phosphate composite cation-exchange material. CTAB: N-cetyl-N,N,N-trimethylammonium bromide.

S. No.	Mixing Volume Ratios (*v*/*v*)		Graphene (mg) Dispersion in 1 mmol CTAB	Appearance of Beads after Drying	Na^+^ Ion Exchange Capacity (meq·dry·g^−1^)
	0.1 M ThPO_4_·5H_2_O in 1 M HNO_3_	2 M H_3_PO_4_			
S-1	1	1	50	Light Blue	1.48
S-2	1	1.5	100	Light Blue	1.56
S-3	1	2	150	Light Blue	1.56
S-4	1	2.5	200	Light Blue	1.56

**Table 2:** A comparison of ion-exchange capacity of previously reported lead-selective composite materials with the present study.

S. No.	Ion-Exchanger	Ion-Exchange Capacity (meq·dry·g^−1^)	Reference
1	EDTA-stannic (IV) iodate	0.74	[34]
2	Polypyrrole Th(IV) phosphate	1.56	[23]
3	Polyaniline Sn(IV) tungstophosphate	1.10	[35]
5	Graphene Thorium(IV) phosphate	1.56	Present Study

**Table 3:** Ion-exchange capacity of various exchanging ions on a hybrid of graphene Th(IV) phosphate composite cation-exchanger.

Exchanging Ions	pH of the Metal Solution	Ionic Radii (Å)	Hydrated Ionic Radii (Å)	I.E.C. (meq·dry·g^−1^)
Li^+^	6.7	0.68	3.40	1.30
Na^+^	6.7	0.97	2.76	1.56
K^+^	6.8	1.33	2.32	1.70
Mg^2+^	6.5	0.78	7.00	1.20
Ca^2+^	6.5	1.06	6.30	1.34
Sr^2+^	6.3	1.27	-	1.50
Ba^2+^	6.3	1.43	5.90	1.68

**Table 4:** Effect of temperature on ion-exchange capacity (IEC) of graphene Th(IV) phosphate composite cation-exchanger on heating.

Heating Temperature (°C)	Appearance (Color)	Weight Loss (%)	Na^+^ Ion-Exchange Capacity (meq·dry·g^−1^)	% Retention of I.E.C.
50	Light Blue	-	1.56	100
100	Light Blue	9.0	1.56	100
150	Storm Grey	11.0	1.40	90.03
200		12.0	1.25	80.12
250	Charcoal grey	12.5	1.08	69.59
300	Mist Grey	13.5	1.0	64.10
350	Mist Grey	15.0	0.90	60.33
400	Mist Grey	15.0	0.88	56.56
450	Mist Grey	15.5	0.82	53.10

**Table 5:** K_d_-values of some metal ions on graphene Th(IV) phosphate column in different solvent systems.

Solvents Metal Ions	K_d_-Values (mL/g)									
	Demineralized Water (DDW)	10^−2^ M HClO_4_	10^−1^ M HClO_4_	1 M HClO_4_	10^−2^ M HNO_3_	10^−1^ M HNO_3_	1 M HNO_3_	10^−2^ M HCl	10^−1^ M HCl	1 M HCl
Mg(II)	95	46	28	17	30	26	22	25	20	14
Ca(II)	134	140	107	83	112	94	81	104	85	67
Cd(II)	80	85	69	45	92	77	52	81	63	47
Pb(II)	560	640	593	548	580	523	470	500	430	380
Co(II)	135	155	128	117	137	123	115	140	117	104
Hg(II)	380	470	426	377	390	362	284	360	337	246
Mn(II)	75	87	71	46	73	56	37	78	52	33
Sr(II)	127	133	114	92	167	142	109	125	103	72
Cu(II)	66	57	39	28	62	45	32	50	31	26
Ba(II)	110	116	94	81	100	86	71	110	93	79

**Table 6:** Some binary separations of metal ions achieved on graphene Th(IV) phosphate.

Separation Achieved	Amount Loaded (mg)	Amount Found (mg)	% Error	Eluent Used	Volume of Eluent (mL)
Cu(II), Pb(II)	1.2710, 4.1440	1.2616, 4.1440	−0.74, 0.00	1 M HCl, 1 M HCl	50, 60
Ba(II), Pb(II)	2.7460, 4.1440	2.7246, 4.1440	−0.80, 0.00	1 M HNO_3_, 1 M HCl	60, 60
Cd(II), Pb(II)	2.2482, 4.1440	2.2257, 4.0881	−1.00, +1.35	1 M HClO_4_, 1 M HCl	50, 60
Ca(II), Pb(II)	4.7230, 4.1440	4.6777, 4.1179	+0.96, −0.63	1 M HCl_,_ 1 M HCl	60, 60
Sr(II), Pb(II)	1.7524, 4.1440	1.7121, 4.1440	−2.30, 0.00	1 M HCl, 1 M HCl	50, 60

**Table 7:** Selective separations of Pb^2+^ ions from a synthetic mixture of Pb^2+^, Ni^2+^, Cd^2+^, and Mg^2+^ ions on a column of graphene Th(IV) phosphate.

S. No.	Amount Loaded (mg)	Amount Found (mg)	Recovery (%)	Error (%)	Volume of Eluent (0.1 M HCl) Used (mL)
1	2.0720	2.0447	98.68	−1.32	50
2	4.1440	4.1150	99.30	−0.70	60
3	6.2160	6.0850	97.89	−2.11	65
4	8.2880	8.1390	98.20	−1.80	75
